# PURA-ECHO: Analysis of the evolution of Ultrasound utilization in austere health care settings in Costa Rica’s Geographic Empanelment Model

**DOI:** 10.1186/s12873-026-01522-x

**Published:** 2026-03-24

**Authors:** Sean Emerson Treacy Abarca, Marisela Aguilar, Natalia Padilla, Timothy Batchelor, Kenton L. Anderson, Nicholas Ashenburg

**Affiliations:** 1 Department of Emergency Medicine, University of California Los Angeles, 1100 Glendon Avenue, Suite 1200, Los Angeles, CA 90024 USA; 2 Department of Pediatrics, Olive View-UCLA Medical Center, Sylmar, USA; 3 Department of Molecular & Cellular Biology, University of Arizona, 1007 E. Lowell Street, Tucson, Arizona 85721 USA; 4 Instituto Costarricense de Equidad de Salud, Menlo Park, USA; 5 Department of Emergency Medicine, Stanford University, 500 Pasteur Drive, Stanford, 94305 USA

**Keywords:** Point of care Ultrasound, GIS, Underserved, Rural, Emergency medicine

## Abstract

**Background:**

Geographic empanelment, a model in which patients are assigned to healthcare facilities based on defined catchment areas, has been adopted by Costa Rica’s national health system (Caja Costarricense de Seguro Social, CCSS) to promote equitable distribution of healthcare resources. Point-of-Care Ultrasound (POCUS) and radiology-based consultative ultrasound have become an increasingly important diagnostic tool in emergency and primary care, particularly in resource-limited settings. However, little is known about how geographic and system-level factors influence ultrasound adoption across diverse care environments. This study evaluates temporal and geographic patterns of ultrasound utilization, including both POCUS and radiology-based (consultative) ultrasound, within the CCSS following the implementation of geographic empanelment.

**Methods:**

The POCUS Utilization in Resource Austerity – Echocardiography (PURA-ECHO) study is a retrospective review of CCSS administrative data comparing ultrasound utilization across rural and urban Emergency Departments and urgent care clinics from 2006 to 2022. The primary outcome was the change in the number of POCUS and consultative ultrasound exams performed over time by geographic classification. Geospatial mapping was used to visualize topographical trends in ultrasound use, and linear regression models assessed the association between distance from urban referral centers and ultrasound adoption in rural facilities.

**Results:**

Rural ultrasound utilization demonstrated a highly significant increasing trend from 2006 to 2022 (Mann-Kendall τ = 0.927, *p* < 0.001), with mean exams per site increasing from 485 [SD 341] to 1,101 [SD 953]. Urban centers showed no significant change over the same period (τ = -0.162, *p* = 0.393), with mean exams per site remaining stable at 2,575 [SD 1,826] in 2006 and 2,652 [SD 1,356] in 2022. The expansion in ultrasound utilization was driven primarily by rural centers, which increased total exam volume 3.2-fold while urban capacity remained stable. Geographic distance from the tertiary training center did not significantly predict ultrasound adoption rates (R² = 0.09, *p* = 0.39), indicating equitable technology diffusion across rural regions regardless of proximity to urban centers.

**Conclusion:**

Ultrasound utilization expanded markedly across Costa Rica’s national health system following geographic empanelment, with rural centers demonstrating the most rapid growth. The lack of association between distance from urban centers and adoption rates suggests that the EBAIS infrastructure successfully facilitated equitable ultrasound diffusion across all rural regions. These findings suggest that geographic empanelment models can effectively strengthen both POCUS and radiology-based ultrasound capacity in geographically isolated regions, enhancing diagnostic equity and access within resource-limited healthcare systems.

## Introduction

Ultrasonography, including Point-of-Care Ultrasound (POCUS), has revolutionized the practice of medicine worldwide, allowing for rapid and accurate diagnosis and treatment of various medical conditions [1–3]. The portability, ease of use, and cost effectiveness of ultrasound have made it an increasingly popular diagnostic tool, particularly in resource-limited settings where traditional imaging techniques may not be available [4, 5]. In these resource-limited settings, short-term ultrasound training programs meant to rapidly and reliably disseminate ultrasound skills and knowledge have also proven effective in global health literature [6–8]. However, limited information exists on how concerted efforts to harness the benefits of ultrasonography within resource-austere health systems develop temporally and geographically in a national health system.

Geographic empanelment is a relatively underutilized strategy to address the need for primary health care that tackles underlying social, economic, and political causes of illness. The World Health Organization has codified these efforts into the Declaration of Astana to describe priorities for future public health systems to address these three core causes of poor health [9–11]. Early adopters of this global standard include Costa Rica, Thailand, Brazil, Cuba, and Ghana [11]. Costa Rica’s system uniquely couples geographic empanelment with efforts to strengthen primary health care in line with the Declaration of Astana’s framework. Prior geospatial analyses of Costa Rica’s empanelment model have shown that such a coupling provides equitable access to care across time and geography [12]. Costa Rica’s efforts began with the creation of the EBAIS health system in 1994, with early adoption of point-of-care and consultative ultrasound in the mid to late 2000s [13].

Costa Rica’s national health system may therefore serve as a valuable model in understanding how concerted efforts to increase access to both consultative ultrasound and POCUS in resource-austere settings may progress over time and across different geographic regions (rural versus urban centers) in Emergency Departments and urgent care settings. Specifically, Costa Rica’s health system guarantees universal coverage through a model of geographic empanelment of their health services. This unique and understudied system may serve as a model for the implementation of new technologies such as ultrasonography in rural areas [14]. Geographic empanelment is a health system model in which populations are assigned to specific healthcare facilities or providers based on their place of residence. Simultaneously, health system resources are allocated based on geographic access within empanelment boundaries. This approach promotes equitable access to care, facilitates continuity of services, and allows for better resource allocation and population health management [15, 16]. To achieve geographic health equity through geographic empanelment, Costa Rica established Equipos Básicos de Atención Integral de Salud (EBAIS) in 1995. EBAIS teams are composed of a doctor, nurse assistant, and medical clerk assigned to rural regional centers [17]. All Costa Rican citizens are assigned to an EBAIS team through geographic empanelment, which promotes access and continuity of care [18].

Our study, POCUS Utilization in Resource Austerity – Echocardiography (PURA-ECHO), examined how nationwide ultrasound utilization in Costa Rica changed across time and geography among health practitioners in Costa Rica’s resource-austere national health system, the Caja Costarricense de Seguro Social (CCSS). To study the effect of a geographic empanelment healthcare model on the dissemination of ultrasonography utilization, we used geographic information systems to overlay national trends of ultrasound utilization over time and geography. Prior studies have used geospatial analysis to glean useful knowledge, such as how urban health centers are more likely to utilize POCUS compared to their rural counterparts in well-funded health systems or how availability of advanced ultrasonography techniques varies by region [19–22]. However, no study to date has investigated the longitudinal development of ultrasonography within a geographic empanelment model such as Costa Rica’s.

In the CCSS system, the definition of an “ultrasound-capable” center varies by setting. Some rural sites are small two-room clinics with a single shared ultrasound machine used for both bedside and consultative studies, while others have slightly larger setups with shared equipment across departments. In contrast, large regional and tertiary training hospitals typically mirror U.S. models, maintaining separate and distinct machines for POCUS and consultative ultrasound. This variation reflects the heterogeneous infrastructure of the national system and informed our inclusion of all ultrasound-capable centers in the analysis. In parallel with the expansion of ultrasound-capable facilities, the CCSS health system supported dissemination of ultrasonography through a combination of equipment allocation and workforce deployment. While no single national mandate governed ultrasound distribution, machines were progressively introduced into rural EBAIS clinics and regional centers as part of broader infrastructure strengthening efforts. Additionally, newly graduated physicians in Costa Rica complete a mandatory two-year social service period in rural settings following residency, ensuring regular deployment of clinicians with foundational ultrasound training. Together, the coordinated expansion of equipment, trained personnel, and existing geographic empanelment infrastructure created the conditions under which ultrasound utilization could expand across rural regions.

The objectives of this study were to: (1) understand how both POCUS and consultative ultrasonography utilization developed temporally and geographically within Costa Rica’s CCSS geographic empanelment healthcare model via geospatial analysis; and (2) evaluate the effects of geographic empanelment on POCUS and ultrasound utilization between urban healthcare centers and more resource-limited rural centers. Our primary outcome was the difference in number of POCUS and ultrasound exams performed between rural and urban healthcare centers in Costa Rica at the beginning and end of the study period.

## Materials and methods

### Study design

This study was exempted from review by the Institutional Review Board (IRB) at Stanford University. Publicly available data was retrieved from the CCSS electronic archives [23]. Data was extracted from the CCSS archives database, which includes information on the utilization of total ultrasound modalities, including point-of-care ultrasound (POCUS) and consultative ultrasound from 2006 to 2022 in Costa Rica within Emergency Departments or urgent care centers. Data extracted included the number and location of ultrasound-capable Emergency Departments or urgent care centers and the total number of exams performed per center and per patient visit. All emergency department and urgent care centers in the CCSS system operating in 2006 and those established in the interim period to 2022 were included. Data was extracted by a single investigator using a standardized data extraction tool. The study period of 2006–2022 was selected because reliable national data collection on both consultative and point-of-care ultrasound utilization began in 2006, and 2022 represented the most recent complete year of available CCSS data.

In Costa Rica’s national health system, the CCSS electronic records do not distinguish between POCUS and consultative ultrasound studies. Particularly in rural settings, these modalities often overlap in practice—ultrasound exams are frequently performed and interpreted directly by the primary physician at the point of care, with radiologist review occurring days later or not at all. Because of this operational and reporting overlap, and to maintain data integrity, both POCUS and consultative ultrasound exams were analyzed together as a composite measure of total ultrasound utilization across sites.

This study followed the Strengthening the Reporting of Observational Studies in Epidemiology (STROBE) guidelines. The study protocol adhered to the ethical standards outlined in the Declaration of Helsinki.

### Analysis

Mann-Kendall trend tests were used to assess monotonic trends in ultrasound utilization over the study period (2006–2022), with statistical significance set at *p* < 0.05. Kendall’s tau (τ) coefficient was calculated to indicate the strength and direction of temporal trends. Linear regression analysis was performed to quantify the rate of change in total ultrasound exams over time for rural centers, with results reported as slope estimates with 95% confidence intervals and R² values. To determine if distance from the urban tertiary care center with the highest total ultrasound utilization (both POCUS and consultative exams combined) influenced the percent increase of ultrasound utilization among rural underserved hospitals, a separate linear regression was conducted. Hospital Calderón Guardia, the primary tertiary teaching hospital with the highest ultrasound utilization, was used as the single reference point for all distance calculations. Healthcare centers located more than 50 km from Hospital Calderón Guardia were classified as rural, while those within 50 km were considered urban; this threshold generally encompasses the edge of urban sprawl surrounding Costa Rica’s major population centers. Distances were calculated using current available overland travel routes rather than straight-line geospatial distances to more accurately reflect real-world travel accessibility and geographic isolation.

**Fig. 1:**
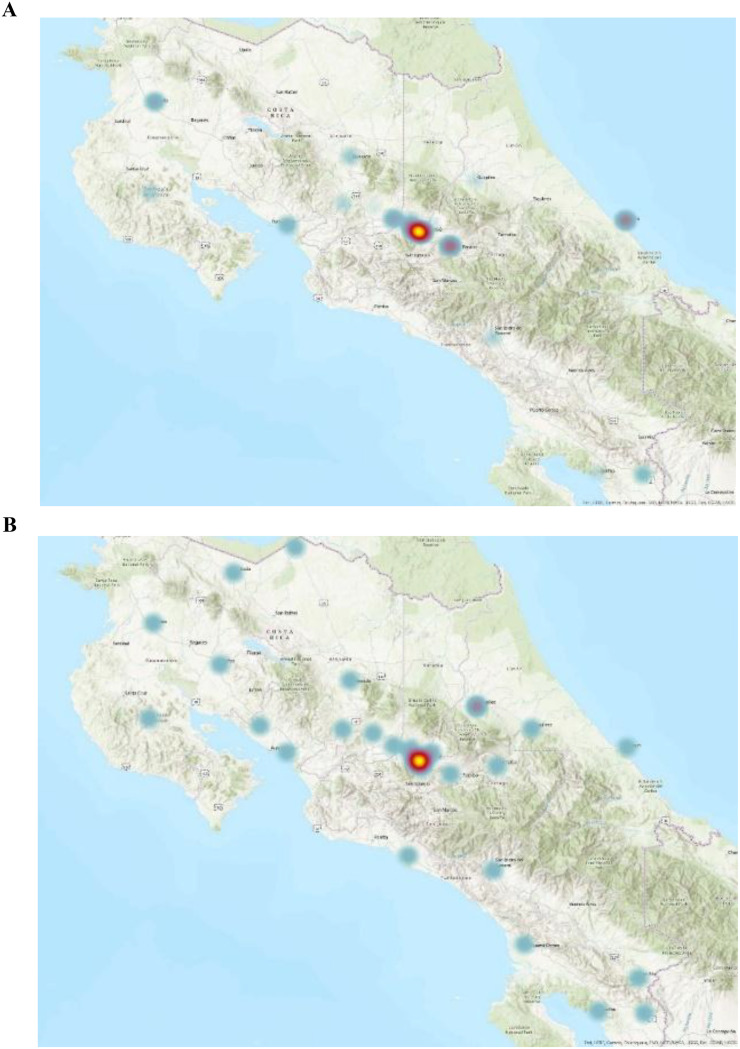
Heat map distribution of total ultrasound exam utilization (including point-of-care and consultative ultrasound) within Emergency Department and urgent care settings in 2006 and 2022. Denser utilization corresponds to warmer colors, while sparser utilization corresponds to cooler colors. New areas of ultrasound activity are represented by emergent regions of heat signal over time

For Geospatial Mapping (GIS), the unique latitude and longitude coordinates based on reported location of the exam were used. Data were analyzed using STATA version 16.0 (Stata Corp LLC, College Station, TX, USA). On STATA, composite aggregation of all ultrasound studies for each health center were tabulated and used to generate an Earth Systems Research Institute (ESRI) point data file. To provide a visual representation of the growth of ultrasound utilization in Costa Rica from 2006 to 2022, the CCSS dataset was overlaid using ArcGIS Pro 2.8 (Earth Systems Research Institute, Redlands, CA, USA) mapping software to generate a topographical representation of ultrasound utilization in different regions of Costa Rica between 2006 and 2022. A heat density map compares the utilization of ultrasound in 2006 and 2022 (Fig. 1).

## Results

The total number of healthcare centers serving rural areas, as defined by the CCSS, increased from 9 in 2006 to 14 in 2022, while the total number of ultrasound-capable centers in urban metropolitan areas increased from 10 in 2006 to 11 in 2022. The number of ultrasound exams performed at these centers is presented in Table 1.

**Table 1:** Total ultrasound utilization (including point-of-care and consultative ultrasound) by care center type, 2006–2022

Type of Care Center	Ultrasound-Capable Centers 2006 *N* (%)	Ultrasound-Capable Centers 2022 *N* (%)	Total Ultrasound Exams 2006 *N* (%)	Total Ultrasound Exams 2022 *N* (%)
Rural Centers	10 (50)	14 (56)	4,847 (16)	15,35 (34)
Urban Centers	10 (50)	11 (44)	25,750 (84)	29,171 (66)
Total	20 (100)	25 (100)	30,597 (100)	44,524 (100)

Time-series analysis revealed distinct utilization patterns between rural and urban centers over the study period. Rural health centers demonstrated a highly significant increasing trend in mean ultrasound exams per site (Mann-Kendall τ = 0.927, *p* < 0.001), with utilization increasing from 485 [SD 341] exams per site in 2006 to 1,101 [SD 953] exams per site in 2022, representing a 2.3-fold increase (Table 2). Linear regression analysis confirmed this upward trend, with rural centers averaging an increase of 713 exams per year (95% CI: 623–803, R² = 0.94). In contrast, urban health centers showed no significant trend over the study period (Mann-Kendall τ = -0.162, *p* = 0.393), with mean exams per site remaining stable at 2,575 [SD 1,826] in 2006 and 2,652 [SD 1,356] in 2022, representing only a 3% change (Table 2). This differential growth pattern indicates that the expansion in ultrasound utilization was driven primarily by rural centers, with total exam volume increasing 3.2-fold in rural areas while urban capacity remained stable.

**Table 2:** Number of POCUS exams performed at rural and urban centers in 2006 and 2022

Mean number of exams	2006 Mean [SD]	2022 Mean [SD]	*p*-value* (Mann-Kendall τ)
**Per site**			
Rural Centers	485 [341]	1,101 [953]	*p* < 0.001 (τ = 0.93)
Urban Centers	2,575 [1826]	2,652 [1356]	*p* = 0.393 (τ = -0.16)

SD=standard deviation * Mann-Kendall trend test for monotonic trend, 2006–2022. τ = Kendall’s tau coefficient

**Fig. 2:**
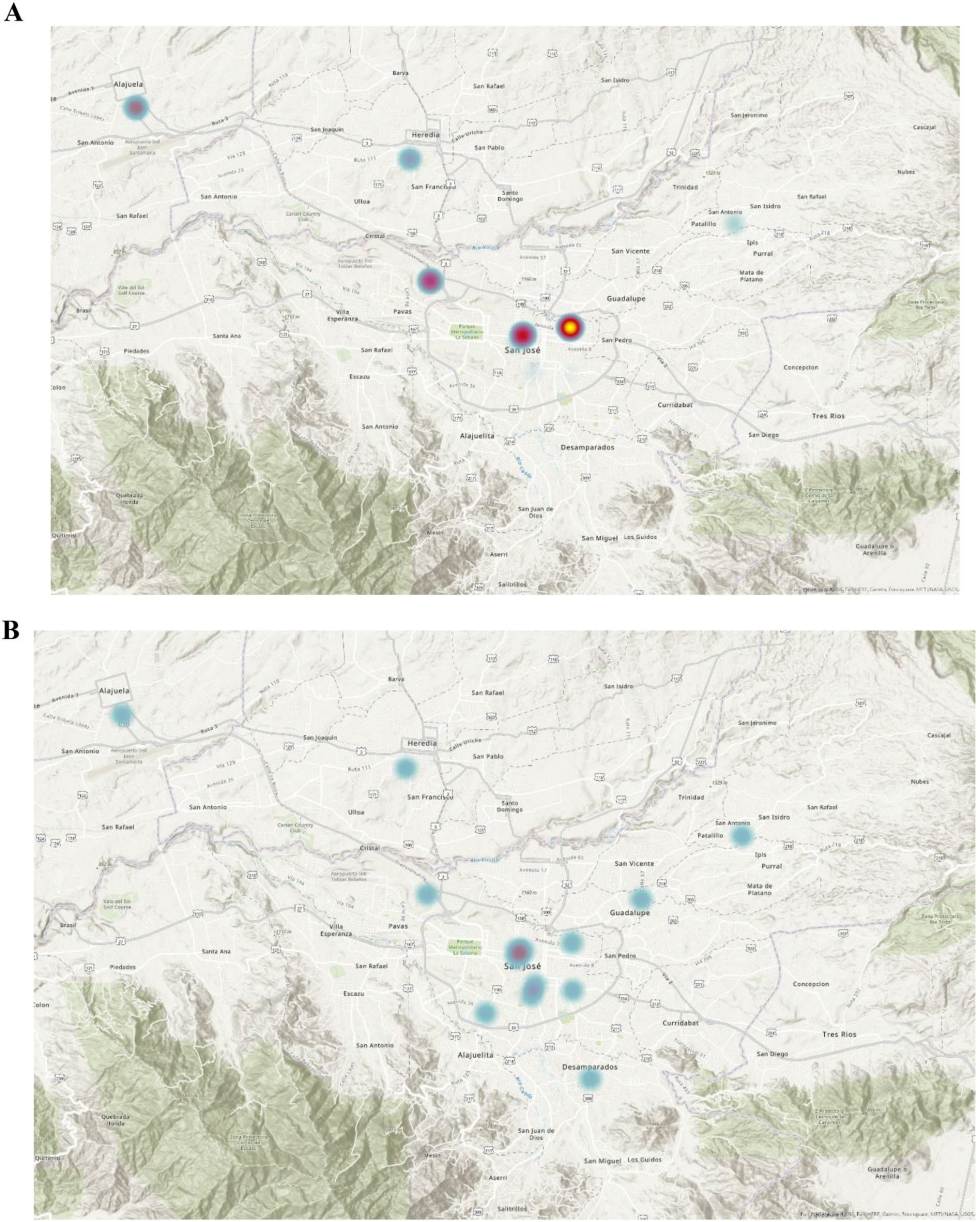
Heat map distribution of total ultrasound utilization (including point-of-care and consultative ultrasound) within Emergency Department and urgent care settings in 2006 and 2022 in the Greater San José Metropolitan Area. Denser utilization corresponds to warmer colors, while sparser utilization corresponds to cooler colors

**Fig. 3:**
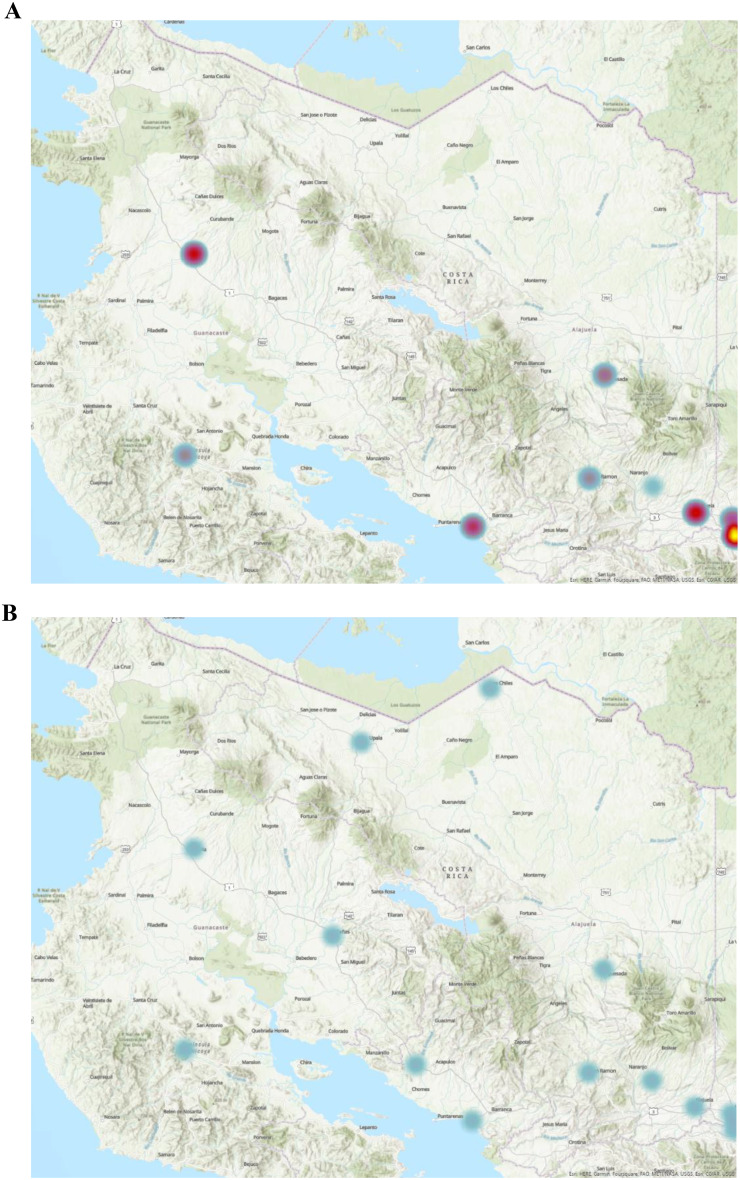
Heat map distribution of total ultrasound utilization (including point-of-care and consultative ultrasound) within Emergency Department and urgent care settings in 2006 and 2022 in the Nicoya region bordering Nicaragua. Denser utilization corresponds to warmer colors, while sparser utilization corresponds to cooler colors

**Fig. 4:**
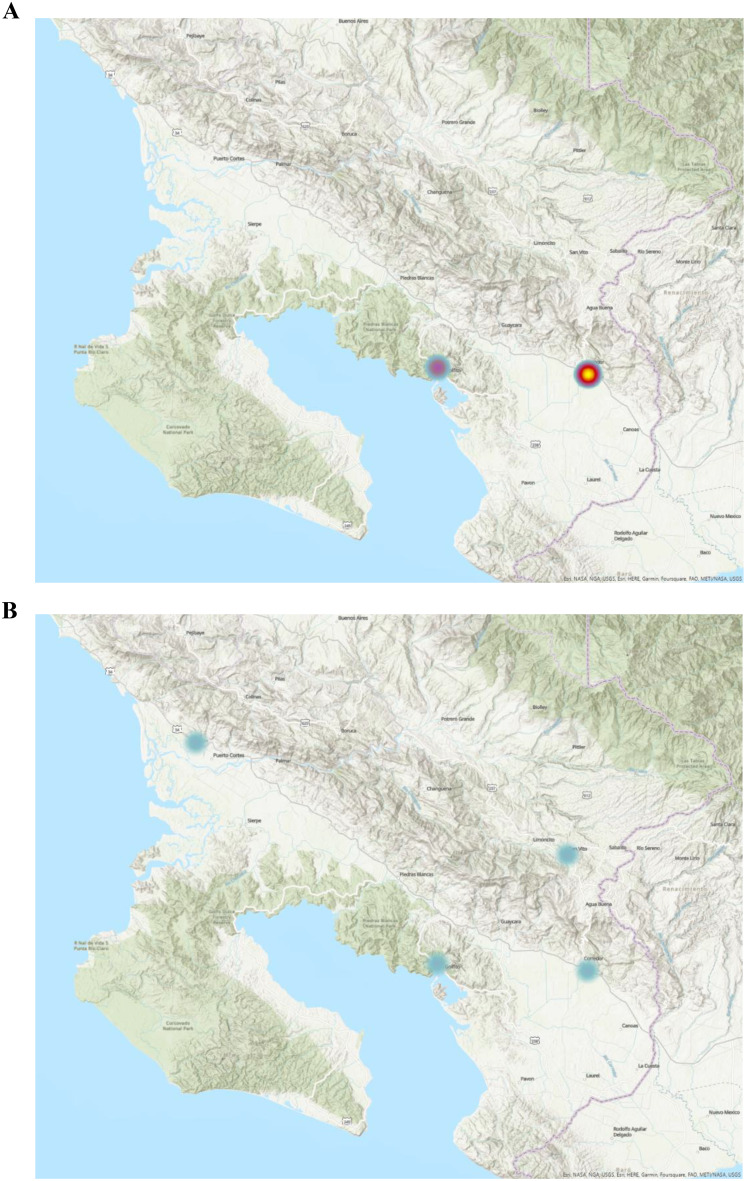
Heat map distribution of total ultrasound utilization (including point-of-care and consultative ultrasound) within Emergency Department and urgent care settings in 2006 and 2022 in the southern Guanacaste region bordering Panama. Denser utilization corresponds to warmer colors, while sparser utilization corresponds to cooler colors

The map displays the spatial distribution of ultrasound utilization, with areas of high utilization represented by warmer colors and areas of low utilization represented by cooler colors. On a country-based macro level, 10 new ultrasound-capable centers were established from 2006 to 2022 (Fig. 1). The densely populated metropolitan San Jose Valley increased access from 6 centers to 11 (Fig. 2). As the number of ultrasound-capable centers increased, the proportion of total ultrasound studies conducted at urban tertiary care centers in the San Jose Valley decreased (Fig. 2). The rural Nicoya region in northern Costa Rica experienced an increase in ultrasound-capable centers, increasing from 2 to 6 centers during the study period (Fig. 3). The second rural area with an increase in ultrasound utilization is the southern Guanacaste region bordering Panama, which doubled from 2 ultrasound-capable centers in 2006 to 4 by 2022 (Fig. 4). As the number of service centers in Guanacaste increased, ultrasound utilization at regional centers decreased while ultrasound utilization conversely increased at rural centers (Fig. 3).

**Fig. 5:**
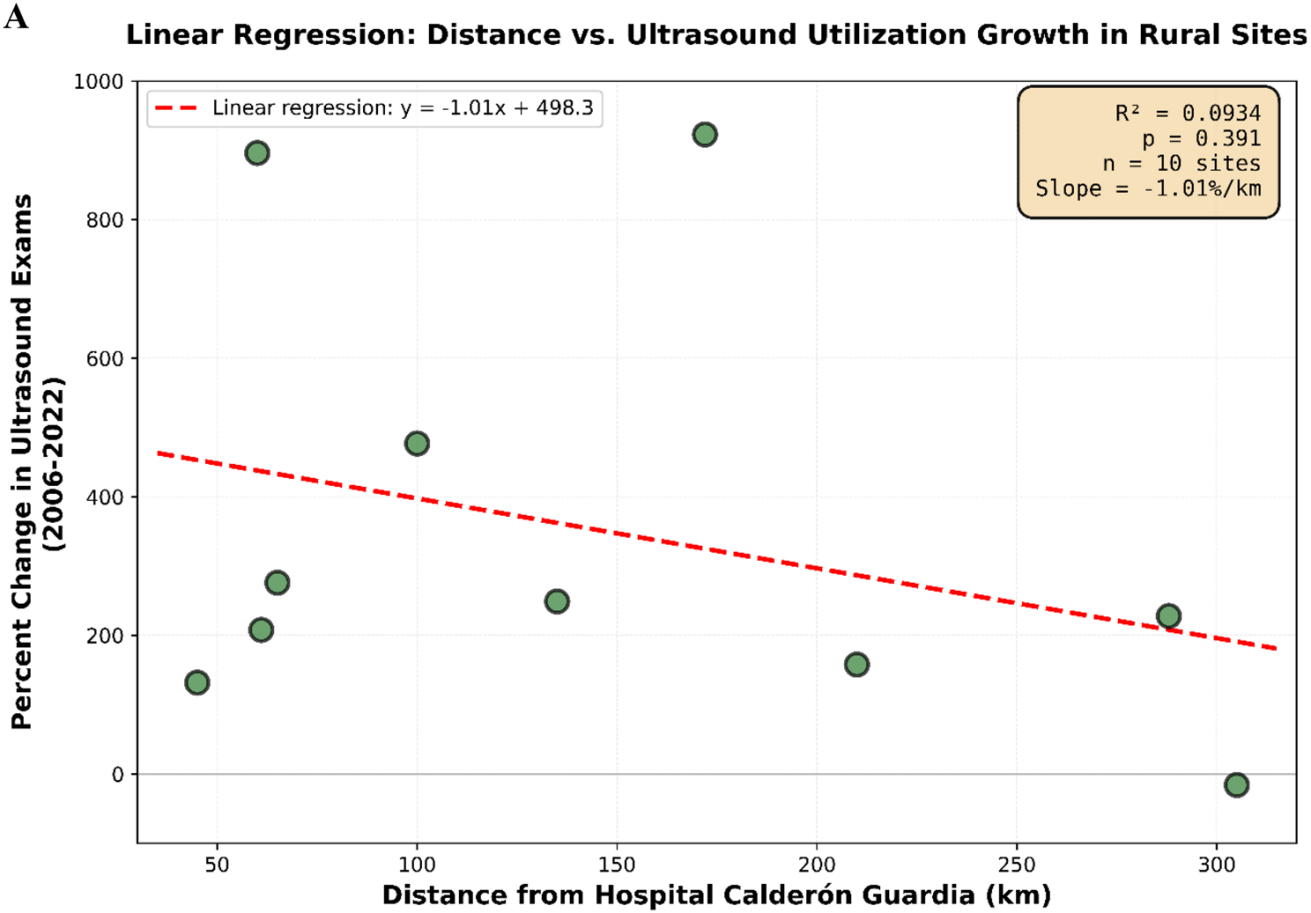
Linear regression analysis of new or expanding rural health centers conducting total ultrasound examinations (including point-of-care and consultative ultrasound) in Costa Rica, evaluating the association between distance (kilometers) from the tertiary care hospital with the highest ultrasound utilization and the percent increase in total ultrasound utilization from 2006 to 2022

Linear regression analysis examined whether distance from Hospital Calderón Guardia (the tertiary teaching hospital with the highest total ultrasound utilization) was associated with the magnitude of ultrasound expansion in rural facilities. No statistically significant relationship was observed between geographic distance and percent increase in ultrasound utilization (R² = 0.09, *p* = 0.39), indicating that ultrasound adoption in rural centers occurred independently of proximity to the tertiary training center (Fig. 5). This finding suggests that the EBAIS infrastructure enabled equitable technology diffusion across all rural regions, rather than creating a gradient of adoption based on distance from urban centers.

## Discussion

The PURA-ECHO study is the first to longitudinally track the development of ultrasonography—both consultative and POCUS modalities—within a national health system serving rural and geographically isolated populations in a resource-limited setting. Our study finds that integrating ultrasound into a geographic empanelment model is an effective means of disseminating the modality to geographically isolated regions within resource-austere health systems. Further, geospatial analysis within ultrasonography literature is an underutilized tool that was effective in identifying the success of the EBAIS geographic empanelment system at increasing ultrasound utilization. The PURA-ECHO study is also the first to investigate how concerted integration of POCUS into geographic empanelment may increase ultrasound utilization in a resource-austere setting. The findings highlight the utility of geospatial analysis in understanding how ultrasonography is utilized and how it can expand diagnostic capacity in rural and underserved regions.

In the PURA-ECHO study, geospatial analysis was instrumental in highlighting how ultrasonography evolves over time and geography. Using geospatial analysis, we were able to recognize robust adoption of ultrasonography in geographically isolated rural regions of Costa Rica. This is particularly relevant for resource-limited settings where access to healthcare and medical technologies can be challenging in remote areas. In fact, past studies have shown that in the United States a clear inverse relationship exists; as populations become more rural, ultrasound usage typically decreases likely due to key sociodemographic and economic variables [24]. Our geospatial analysis highlights the regional effect and benefits of developing robust rural ultrasound capability, which could be translated to care in high-income countries such as the United States with large rural populations.

Time-series analysis revealed that the expansion in ultrasound utilization was driven primarily by rural centers, which demonstrated a highly significant increasing trend (Mann-Kendall τ = 0.927, *p* < 0.001) with mean exams per site increasing 2.3-fold (from 485 to 1,101 exams per site) from 2006 to 2022. In contrast, urban centers showed no significant trend over the study period (τ = -0.162, *p* = 0.393), with per-site utilization remaining stable. This differential growth pattern suggests that strategies to increase ultrasound utilization in rural areas can be implemented without compromising existing urban healthcare capacity. The PURA-ECHO study demonstrated that as ultrasonography capability developed in rural EBAIS clinics, the proportion of total ultrasonography scans performed at already strained regional referral hospitals decreased across Costa Rica. This pattern was evident in the greater San Jose Valley metropolitan area, the northern Nicoya province, and the southern Guanacaste region. In all three areas, as smaller rural health centers gained ultrasound capacity, reliance on regional nodes of high utilization diminished. These shifts were not solely the result of distributing ultrasound machines but also reflected the deliberate expansion of trained personnel and infrastructure described in the geographic empanelment framework. Within this system, new physicians complete a mandatory two-year social service period in rural clinics following residency, ensuring a steady deployment of clinicians with core POCUS training. Together, the combination of trained personnel, thoughtfully allocated equipment, and existing infrastructure likely drove the observed increase in rural ultrasound utilization, while utilization in urban centers remained stable.

While the CCSS did not implement a specific decree mandating POCUS integration, the observed expansion appears to have occurred organically through the existing EBAIS infrastructure and its emphasis on equitable resource distribution. Notably, geographic distance from Hospital Calderón Guardia did not significantly predict the magnitude of ultrasound expansion in rural facilities (R² = 0.09, *p* = 0.39). This lack of distance-dependent gradient in adoption rates represents a key strength of the geographic empanelment model, demonstrating that the EBAIS infrastructure successfully facilitated technology diffusion across all rural regions, including those most distant from tertiary training centers. This finding contrasts with typical patterns observed in market-driven healthcare systems, where proximity to academic medical centers often predicts technology adoption. Instead, the EBAIS model’s systematic allocation of trained personnel and resources appears to have overcome geographic barriers, ensuring that even the most remote rural facilities achieved substantial increases in ultrasound utilization. This equitable distribution pattern underscores the potential of geographic empanelment frameworks to reduce healthcare disparities in geographically diverse, resource-limited settings.

The stability of urban ultrasound utilization throughout the study period, contrasted with significant rural growth, provides important evidence for the targeted success of geographic empanelment policies. The lack of significant trend in urban centers (*p* = 0.393) suggests that urban facilities maintained consistent utilization patterns while resources and training were directed toward expanding rural capacity. This finding addresses a key concern in healthcare expansion initiatives: that resource allocation to underserved areas might occur at the expense of existing services. Our data demonstrate that rural ultrasound expansion occurred through genuine capacity building rather than redistribution of urban resources. The increased variability in rural site utilization by 2022 (SD = 953 vs. 341 in 2006) likely reflects the heterogeneity of newly established facilities, ranging from small community clinics to larger regional centers, each serving populations with different healthcare needs and geographic accessibility challenges.

Geospatial analysis highlights the effect of high regional node utilization, likely enabling the spread of new technological modalities like ultrasonography. In Liberia, Costa Rica, the region’s main hospital has had moderate ultrasound utilization since 2006, though only two total centers in the area were capable of ultrasonography. This improved to six centers in 2022, highlighting potential influence from nearby geographic centers of high utilization. The expansion of ultrasound capabilities across multiple regions of Costa Rica, combined with the absence of a distance-dependent adoption gradient, suggests that both regional networks and the national EBAIS infrastructure contributed to equitable technology dissemination.

Future research should revisit these national utilization trends as consultative and POCUS exams become more distinctly captured within the CCSS dataset, allowing for more granular analysis of modality-specific adoption. Beyond Costa Rica, the geographic empanelment framework may serve as a model for improving diagnostic equity in integrated single-payer systems such as the U.S. Indian Health Service and Veterans Health Administration. Investigating the adaptation of empanelment principles within these systems could help determine how structured allocation of both human and technological resources enhances patient access, continuity of care, and diagnostic capacity in underserved populations.

## Limitations

The PURA-ECHO study is an analysis of a single national health system with relatively low per-capita spending on health and human services, which may limit generalizability to other countries with differing healthcare structures. Additionally, while this study sought to characterize both consultative and Point-of-Care Ultrasound (POCUS) utilization, the CCSS dataset does not reliably distinguish between these two modalities. This overlap reflects real-world practice, particularly in rural settings where a single provider may both perform and interpret studies. Although this limits modality-specific analysis, it enhances ecological validity by representing the integrated manner in which ultrasound is used across Costa Rica’s geographically empaneled healthcare system. Finally, we acknowledge that the increasing adoption of ultrasound globally during this period may have also contributed to the trends observed; therefore, the observed growth in utilization cannot be attributed solely to geographic empanelment.

## Conclusions

Our retrospective analysis of the CCSS database highlights a significant increase in ultrasonography utilization across Costa Rica’s rural healthcare centers, driven by the implementation of a Geographic Empanelment Model. Time-series analysis demonstrated a highly significant increasing trend in rural ultrasound utilization (Mann-Kendall τ = 0.927, *p* < 0.001), with mean exams per site increasing 2.3-fold from 485 in 2006 to 1,101 in 2022, while urban centers maintained stable utilization patterns throughout the study period. While the empanelment model was not specifically designed to expand ultrasound services, its broader aim to ensure equitable distribution of both human and physical healthcare resources likely created the structural conditions that enabled ultrasound adoption in underserved regions. Importantly, the lack of association between geographic distance from tertiary training centers and ultrasound adoption rates (R² = 0.09, *p* = 0.39) demonstrates that the EBAIS infrastructure successfully facilitated equitable technology diffusion across all rural regions, including the most geographically isolated areas. The findings demonstrate that targeted efforts to strengthen system-wide access and resource allocation through geographic empanelment can lead to substantial and equitable gains in diagnostic imaging in resource-limited settings. While the study provides valuable insights into the potential of national-level policies to promote equitable healthcare access, further research is warranted to explore how similar models can be adapted to other low-resource settings. Overall, the integration of both consultative and point-of-care ultrasound modalities underlines the real-world applicability of this approach in diverse clinical environments.

## Data Availability

The datasets analyzed during the current study are publicly available from the Caja Costarricense de Seguro Social (CCSS) through its open data portal (https:/www.ccss.sa.cr/datos-abiertos). Additional access to underlying administrative datasets may be obtained from the CCSS upon reasonable request, subject to institutional policies and data use conditions. All analyses in this study were performed using aggregated, de-identified data.

## Declarations

### Author information

SETA is an emergency physician and health systems researcher with academic interests in point-of-care ultrasound, geospatial analysis, and diagnostic equity in resource-limited and geographically isolated settings. The co-authors have clinical and research expertise spanning emergency medicine, global health, and health services research.
